# The Care Dependency of Community-Dwelling Older Adults: A Latent Variable Approach Using Item Response Theory

**DOI:** 10.1093/geroni/igaf122.2468

**Published:** 2025-12-31

**Authors:** Alejandra Marroig, Fernando Massa, Janet Trujillo, Graciela Muniz-Terrera, Sofia Domingorena

**Affiliations:** Universidad de la Republica Uruguay, Montevideo, Montevideo, Uruguay; Universidad de la Republica Uruguay, Montevideo, Montevideo, Uruguay; Universidad de la Republica Uruguay, Montevideo, Montevideo, Uruguay; Ohio University, Athens, Ohio, United States; Universidad de la Republica Uruguay, Montevideo, Montevideo, Uruguay

## Abstract

**Background:**

Older adults living in the community may have functional limitations and require varying levels of assistance to satisfy their care needs. This study aimed to assess the individual degree of care needs in a population-based study of community-dwelling older adults. Using a latent variable approach, we assessed the degree of care dependency and studied its association with socioeconomic and health characteristics.

**Methods:**

We used data on adults aged 60 years and older from the Uruguayan population-based study Encuesta Longitudinal de Protección Social (ELPS) (N = 5102). To assess care needs, we considered 11 Activities of Daily Living and the self/proxy-reported information about the help needed to perform these activities. Using an Item Response Theory model, we estimated the dependency score (DS) used to measure the degree of care dependency. Then, we applied linear regression models to analyze the association between DS and age, sex, education, income, and health characteristics.

**Results:**

The results showed differences in the ADLs in which older adults with a low level of dependency and those with a high level of dependency needed assistance. Additionally, older women (p = 0.002) with multimorbidity (p < 0.001) and individuals with lower levels of education (p = 0.006) had the highest degree of care dependency.

**Conclusions:**

Social and healthcare public policies will benefit from considering the socioeconomic and health differences of older adults. The degree of care dependency should be considered for the design of services providing support to older adults with care dependency living in the community.

